# Factors affecting social isolation among the young adults in South Korea: A cross-sectional analysis

**DOI:** 10.3389/fpubh.2022.979136

**Published:** 2022-09-06

**Authors:** Soo-Bi Lee, Yerim Shin, Yebin Jeon, Seohyun Kim

**Affiliations:** Department of Social Welfare, Jeonbuk National University, Jeonju-si, South Korea

**Keywords:** loneliness, social isolation, young adults, predictor, cross-sectional analysis

## Abstract

The recent increase in lonely deaths among young people has emphasized the emergence of social isolation as a social problem. This study investigated the issue of social isolation by examining multidimensional factors that affect social isolation and evaluating the subjective and objective dimensions of young adults. Data for this study were collected for 8 days (February 7–14, 2022) through an online questionnaire survey by a professional survey agency, based on proportional allocation of the young adult population (age 19–39 years); data from 521 young adults were analyzed. Regression analysis was performed using SPSS to identify multidimensional factors (relative deprivation, future outlook, depression, self-esteem, social skills, experience of violence, and online activity) that affect social isolation and showed that: (1) among sociodemographic characteristics, higher age and unemployment were associated with greater social isolation; (2) sociopsychological characteristics, such as higher levels of relative deprivation and depression, more negative future outlook, and lower self-esteem, all correlated with greater social isolation; and (3) among relationship characteristics, lower social skills and a stronger history of experiencing violence were linked to greater social isolation. These results highlight the need for a customized support system at the national level that takes into account the developmental tasks of young adults as a preventive strategy to solve the problem of social isolation of young adults and to devise various strategies to provide them with mental health services.

## Introduction

The social distancing that has been implemented over the past 2 years during the coronavirus disease (COVID-19) pandemic has resulted in relationship problems, such as isolation, loneliness, and disconnectedness, among people worldwide. Hertz pointed out that the problem of isolation, that is, feelings of loneliness, was a social problem that preceded the COVID-19 pandemic and raised concerns about the escalation of this social problem into a more serious public health issue, which has been subsequently accelerated by the COVID-19 pandemic (1). The concept of social isolation has multifaceted implications in various aspects, including subjective isolation and objective state of isolation. Social isolation can be understood in two dimensions: external isolation, represented by disconnection of social networks and the lack of contact with or support from others, and an internal isolation, represented by emotional loneliness or a negative internal reaction that arises from the absence of informal support relationships (2–4). When exploring social isolation separately, objective and subjective isolation can be considered as a mutually differentiated but correlated concept (5–7) in that the level of loneliness and social isolation does not subside by forming personally meaningless relationships with others even if the frequency of social interactions and relationships with others is high (8). Therefore, efforts to understand social isolation should embrace not only the state of absence of quantitative and qualitative interpersonal relationships, but also the individually perceived feelings of social isolation and loneliness (2, 9–13).

Social isolation is associated with an individual's physical and mental health, as well as with various social issues. Social isolation is a risk factor for depression and mental illness and reduces the quality of life and wellbeing (14–16). Furthermore, socially isolated people are more likely to lose control over their lives, have difficulty receiving help from others, and face an increased risk of loneliness, poverty, and mortality (17, 18).

With the recent increase in lonely death and suicide rate among young adults (19), which are emerging social issues in South Korea, the problem of social isolation among young people has come into the spotlight as a causative factor. In a survey conducted by the National Youth Policy Institute (20), 13.4% of young people responded that they felt isolated from others, and 16.6% experienced acute feelings of loneliness, which made them feel as if they were completely alone in the world. To address this phenomenon, researchers and specialists in various fields have emphasized the importance of understanding the multidimensional social situation and the context in which young people find themselves (21). Currently, a large number of young people in South Korea experience unstable housing and job problems due to the high unemployment rate, debt from student loans, and high housing rates (22, 23). As a result of the economic downturn and disruption of social relationships caused by the COVID-19 pandemic, young people are facing multilayered, aggravated difficulties. Therefore, many young people in South Korea give up on the employment, date, marriage, and interpersonal relationships that they should enjoy during their youth life course, and the number of single-person households has increased.

Young people living in this perceivably modern society find it increasingly difficult to fulfill the societal roles required of the young adults and, when faced with their inability to control their own future, they experience anger, relative deprivation, depression, and helplessness (24–26).

Moreover, relative deprivation negatively affects social relationships, leading to social withdrawal and social isolation (27). Against this background, many young people in modern society lose hope (28) and experience psychological and social isolation (29–32). In particular, increased negative self-image and frustration due to a secluded and isolated life increases helplessness and depression, which will likely result in prolonged social isolation (33).

Meanwhile, some studies have identified depression, self-esteem, poor social skills, and adverse childhood experience (34)—that is, experiences of violence and abuse from friends and family—as causes of social isolation (35–37). Hertz argued that online technology-based activities, such as hyperconnected social media, can further increase loneliness (1). Considering the above aspect, it can be expected that social isolation is closely related to psychological and emotional states at the individual level and negative life experiences, such as impaired social skills due to violence, socioeconomic situation, related hopelessness, and relative deprivation. Thus, it can be construed that young people experience delays in and loss of their social roles and disconnection of relationships due to their internal characteristics, their multidimensional external environmental factors, and the interactions between those factors, all of which lead to social isolation. Therefore, social isolation is caused by diverse and complex factors, either singly or in combination.

Although social isolation can occur at any stage of life (36–38), previous studies have mostly focused on the older population (9, 39–41) and, in view of the absolute number of studies, have not very intensely investigated this concern in the young population. In addition, some of studies have been conducted on social isolation and mental health outcomes since the outbreak of COVID-19 (42–44). However, these studies have focused more on objective quantification of social activities, lack of jobs, and social networks on single dimension (39, 45, 46). As mentioned above, it is important to consider that both external isolation, resulting from a disconnection of relationships and social networks, and internal isolation, resulting from internal disconnection and loneliness, are important aspects of social isolation that can manifest together. Accordingly, this study aimed to identify the multidimensional risk factors for social isolation by considering both the subjective and objective isolation dimensions that affect the young adults.

## Materials and methods

### Data

This study used a proportional allocation method to collect data while considering age (5-year-old unit), regional size (metropolitan/non-metropolitan area), and sex with regard to a population of 19- to 39-year-old male and female young adults nationwide (resident registration data as of January 2022), and an online questionnaire survey was conducted using the panel of “Hankook Research” (a professional survey company). Data were collected for 7 days (February 7–14, 2022), and the sampling error range was ±4.3% (95% confidence interval). Although “young people” refers to those who are in their 20s and 30s in accordance with various criteria that have been specified previously (47, 48), recently, a need has emerged to consider the reality that the transition in life course is being delayed due to prolonged studies and delayed employment among young Koreans (49, 50). Data from 521 respondents who were thus selected were used for analysis. This study was approved by the relevant Institutional Review Board, and the study procedures were undertaken in accordance with the Declaration of Helsinki [JBNU 2022-04-003-006].

### Study-related measures

#### Dependent variables

To measure social isolation when considering both external and internal aspects of isolation, each survey item was rated to obtain a total score, and the total item scores, which were z-standardized for unit standardization, were added to obtain the overall total score, which was utilized for analysis. To measure internal isolation—that is, psychological isolation−11 items suggested by De Jong-Gierveld and Van Tilburg were used (51). The items of this scale include: “There is always someone that I can talk to about my day-to-day problems”; “I miss having a really close friend”; “I experience a general sense of emptiness”; “There are plenty of people that I can lean on in case of trouble”; “I miss the pleasure of company of others”; “I feel my circle of friends and acquaintances is too limited”; “There are many people that I can count on completely”; and “Often I feel rejected.” Each item is rated on a 5-point Likert scale (1 = none of the time, 5 = all of the time), and the total score obtained by adding up the item scores is used for analysis. External isolation, which comprises disconnection from external support systems, social capital, or social skills, was measured using the Lubben Social Network Scale (LSNS)-6 that was originally developed by Seeman and Berkman (52) and subsequently revised into a short form by Lubben et al. (53). The scale consists of six items that include “How many relatives do you see or hear from at least once a month?”; “How many relatives do you feel close to such that you could call on them for help?”; “How many relatives do you feel at ease with that you can talk about private matters?”; “How many of your friends do you see or hear from at least once a month?”; “How many friends do you feel close to such that you could call on them for help?”; “How many friends do you feel at ease with that you can talk about private matters?” Each item is rated on a 6-point Likert scale (0 = one, 5 = nine or more), with a higher total score indicating greater objective isolation. The Cronbach's α in this study was 0.856 for objective isolation and 0.752 for subjective isolation, and that of the overall isolation obtained by standardizing and summing up the scores of internal and external isolation scales was 0.847.

#### Independent variables

Relative deprivation was measured with the scale developed by Kwon (54) and Cha (55) who reconstructed the scales proposed by Crosby (56), Smith and Pettigrew (57), Mishra and Carleton (58), Heo et al. (59), and Lee (60). This scale comprises 14 items that include “Other people seem to enjoy a more plentiful life than I do”; “Other people seem to enjoy more benefits than I do”; “I feel deprived when I compare myself with others”; and “I think it is unfair that I do not enjoy benefits that others do.” Each item was rated on a 5-point Likert scale (1 = strongly disagree, 5 = strongly agree), with a higher total score indicating a higher the level of relative deprivation. The Cronbach's α of the abovementioned scale in this study was 0.857.

The future outlook scale used in this study measures the 5-year future outlook as perceived by an individual and consists of five items: income class, living standard, job stability, career opportunity, and level of happiness. Each item is rated on a 5-point Likert scale (1 = much worse, 5 = much better), and a lower total score is associated with a more negative perception of future. The Cronbach's α of the scale in this study was 0.872.

Depression was measured with the Center for Epidemiologic Studies Depression Scale (CESD)-11, which elicits the feelings of the respondent in the past week. The CESD-11 consists of 11 items, including “I did not feel like eating; my appetite was poor”; “I felt depressed”; “I felt that everything I did was an effort”; and “I felt sad.” Two reverse-coded items were recoded for use. Each item was rated on a 4-point Likert scale (1 = strongly disagree, 4 = strongly agree), which was recoded to a 0-to-3 scale. The total score was used for analysis, where a higher score indicated a higher level of depression. The Cronbach's α of the scale was 0.914.

Self-esteem was measured with Rosenberg's (61) Self-Esteem Scale, which includes items such as “I feel that I am a person of worth”; “I feel that I have a number of good qualities”; “All in all, I am inclined to think that I am a failure”; and “On the whole, I am satisfied with myself.” This scale consists of five positive and five negative items, and the latter were recoded for use in this study. Each item was rated on a 4-point Likert scale (1 = rarely, 4 = always), where a higher total score indicates a higher level of self-esteem. The Cronbach's α of this scale was 0.858.

To measure social skills, we applied the concept proposed by Fleming et al. (62). Ten items were used, including “I make friends easily”; “I resolve problems with friends quite well”; “I understand others' feelings and emotions well”; and “I can control my anger even when things don't go my way.” Each item was rated on a 4-point Likert scale (1 = strongly disagree, 4 = strongly agree), where a higher total score indicates a higher level of social skills. The Cronbach's α was 0.811.

The participants' experience of violence was measured using seven items (swearing and insulting, assault and battery, bullying, extortion, intimidation, sexual harassment and abuse [teasing], and forced errands) based on responses to the question “How often have you experienced the following acts from your peers or older/younger students?” Each item was rated on a 5-point Likert scale (1 = never, 5 = once or twice a week), where a higher total score indicates a higher level of experience of violence. The Cronbach's α was 0.901.

Online activity was measured using the question “How much content do you use for smartphone-mediated communication? Respondents are given three response options: (1) E-mail and messenger (KakaoTalk, Facebook Messenger, Line, and others), (2) Social media (Facebook, Instagram, Twitter, Band, Naver Cafe, and others); and (3) Social meeting apps (small group meeting and others). Each item was rated on an 8-point Likert scale (0 = never, 7 = very often), where a higher total score, was associated with a higher likelihood of the respondent engaging in online activity.

#### Control variables

Sociodemographic variables, including sex, age, residential area, economic activity status, household type, household income, and individual income, were included in the analysis. Sex, residential area, and household type were binary categorized, with “1” assigned to male, capital area, and single-household and “0” to female, non-capital area, and non-single household, respectively. Age and monthly household and individual income were used as continuous variables.

### Statistical analyses

This study was analyzed as follows using SPSS ver 0.25 to verify the research model. First, we performed descriptive statistics and correlation analysis to understand the general characteristics of the subjects. Second, multiple regression analysis was performed to identify factors affecting the social isolation of young people.

## Results

### Sociodemographic characteristics

A frequency analysis was performed to examine the participants' sociodemographic characteristics. Table 1 outlines the analysis results as follows: the cohort comprised 260 men (49.9%), and 261 women (50.1%); the overall mean age was 29.4 years. The economic activity status was distributed among students (96 respondents, 18.4%), employed (265, 50.9%), and unemployed (160, 30.7%). In total of 221 respondents had debt (42.4%), and 300 had no debt (57.6%). The individual income range, in increasing order, was below 1 million won (252, 48.4%), 1–2 million won (85, 16.3%), 2–3 million won (103, 19.8%), and more than 3 million won (81, 15.5%). The household income range, in increasing order, was <3 million won (247, 47.4%), 3 to 6 million won (173, 33.2%), and more than 600 million won (101, 19.4%).

**Table 1:** Participants' sociodemographic characteristics (*N* = 539).

**Category**		**Frequency**	**Percentage**
Sex	M	260	49.9
	F	261	50.1
Residential area	Capital area	242	46.4
	Non-capital area	279	53.6
Household type	Non-single household	260	49.9
	Single household	261	50.1
Economic activity status	Students	96	18.4
	Employees	265	50.9
	Unemployed	160	30.7
Debt (Y/N)	Yes	221	42.4
	No	300	57.6
Personal income (KRW)	<500,000	152	29.2
	500,000–1,000,000	100	19.2
	1,000,000–2,000,000	85	16.3
	2,000,000–3,000,000	103	19.8
	≥3,000,000	81	15.5
Household income (KRW)	<3,000,000	247	47.4
	3,000,000–6,000,000	173	33.2
	≥6,000,000	101	19.4
Continuous variable		M	SD
Age (in years)		29.4	5.67

KRW, Korean won.

### Correlation analysis

Table 2 presents the correlation coefficients between the major variables that were evaluated in this study. The correlation coefficients were all <0.8 and ranged from 0.004 to 0.539; the variance inflation factor did not exceed 10 (range 1.060–2.080) and thus met the criteria and demonstrated that there was no problem of multicollinearity.

**Table 2:** Correlation matrix.

	**1**	**2**	**3**	**4**	**5**	**6**	**7**	**8**	**9**	**10**	**11**	**12**	**13**	**14**	**15**	**16**	**17**
1	1																
2	0.004	1															
3	−0.091*	0.052	1														
4	0.013	−0.339**	−0.067	1													
5	0.078	−0.539**	−0.004	0.218**	1												
6	0.020	−0.108*	−0.117**	0.160**	−0.295**	1											
7	−0.090*	0.007	0.002	−0.095*	−0.070	0.003	1										
8	0.162**	0.022	0.098*	−0.019	0.104*	−0.218**	−0.418**	1									
9	0.073	0.392**	0.152**	−0.272**	−0.316**	−0.372**	0.075	0.378**	1								
10	−0.141**	0.065	0.092*	−0.134**	−0.086	0.061	0.103*	−0.183**	−0.128**	1							
11	0.135**	−0.049	0.040	−0.037	−0.017	0.042	−0.026	0.168**	0.152**	−0.228**	1						
12	−0.034	0.054	0.053	−0.140**	−0.109*	0.105*	0.113*	−0.204**	0.090*	0.437**	−0.217**	1					
13	0.070	−0.003	−0.046	−0.017	0.049	−0.133**	−0.049	0.192**	0.169**	−0.413**	0.330**	−0.626**	1				
14	0.039	−0.175**	0.049	0.025	0.145**	−0.016	−0.033	0.162**	0.069	−0.228**	0.270**	−0.346**	0.492**	1			
15	0.114**	−0.011	−0.054	−0.079	−0.060	0.038	0.049	−0.021	0.046	0.099*	−0.051	0.340**	−0.211**	−0.142**	1		
16	−0.126**	−0.047	0.043	−0.026	0.040	−0.116**	0.062	−0.025	0.031	0.058	0.021	0.029	0.004	0.062	−0.025	1	
17	−0.082	0.207**	−0.031	−0.123**	−0.197**	0.158**	0.110*	−0.251**	−0.115**	0.398**	−0.300**	0.524**	−0.549**	−0.462**	0.219**	−0.083	1

1. Sex, 2. Age, 3. Region, 4. Debt (Y/N), 5. Economic Activity_Students, 6. Economic Activity_Unemployed, 7. Household Type, 8. Monthly Household Income, 9. Monthly Individual Income, 10. Relative Deprivation 11. Socioeconomic Future Outlook, 12. Depression, 13. Self–Esteem, 14. Social Skills, 15. Experience of Violence, 16. Online Activities, 17. Social Isolation

^*^p < 0.05,

^**^p < 0.01.

### Factors affecting social isolation

The results of the regression analysis of factors affecting social isolation are presented in Table 3. Among the sociodemographic characteristics, age (*B* = 0.159, *p* < 0.001) and economic activity status of those who were unemployed (*B* = 0.084, *p* < 0.05) had a significant impact on social isolation. This can be interpreted to mean that higher age is associated with a higher level of social isolation and that unemployed young people are more likely to experience social isolation than young people with jobs. Among the sociopsychological characteristics, social isolation was positively affected by relative deprivation (*B* = 0.119, *p* < 0.01) and negatively affected by socioeconomic future outlook (*B* = −0.082, *p* < 0.05). Depression (*B* = 0.195, *p* < 0.001) and self-esteem (*B* = −0.221, *p* < 0.001) were risk factors that significantly affected social isolation—that is, a higher level of perceived relative deprivation was associated with a greater perceived social isolation, and a more negative socioeconomic future outlook of young people was associated with a higher likelihood of their experiencing social isolation. Similarly, the higher the depression level and the lower the self-esteem, the higher was the social isolation. Among the relationship characteristics, social skills (*B* = −0.177, *p* < 0.001), experience of violence (*B* = 0.058, *p* < 0.1), and online activity frequency (*B* = −0.069, *p* < 0.05) significantly influenced social isolation. Fewer social skills, more experience of violence in the past, and less frequent online activity were associated with a higher risk of social isolation.

**Table 3:** Results of regression analysis.

**Variables**	**B**	**SD**	**B**	**95% CI LB**	**95% CI UB**
(Constant)	1.960	0.862		0.265	3.654
Sex	−0.108	0.118	−0.031	−0.340	0.124
Age	0.049***	0.013	0.159	0.023	0.075
Region	−0.130	0.116	−0.037	−0.358	0.099
Debt (Y/N)	−0.170	0.126	−0.048	−0.418	0.078
Economic activity_students	−0.027	0.209	−0.006	−0.437	0.383
Economic activity_unemployed	0.324*	0.162	0.084	0.006	0.642
Non–single household (Y/N)	0.100	0.130	0.029	−0.155	0.356
Monthly household income	−0.036	0.028	−0.055	−0.090	0.018
Monthly individual income	−0.038	0.043	−0.041	−0.122	0.047
Relative deprivation	0.025**	0.008	0.119	0.009	0.040
Socioeconomic future outlook	−0.044*	0.019	−0.082	−0.081	−0.006
Depression	0.046***	0.011	0.195	0.025	0.068
Self–esteem	−0.066***	0.014	−0.221	−0.093	−0.038
Social skills	−0.072***	0.016	−0.177	−0.103	−0.041
Experience of violence	0.021^†^	0.013	0.058	−0.004	0.046
Online activities	−0.030*	0.015	−0.069	−0.059	−0.002

^*^p < 0.05,

^**^p < 0.01,

^***^p < 0.001,

^†^*p* <0.1.

## Discussion

The results of regression analysis in this study of the multidimensional factors affecting social isolation among young people showed the following associations. First, among the sociodemographic characteristics, higher age and unemployment were identified as factors that increased social isolation. This can be comprehended in view of the traditional developmental tasks required of the young adults, wherein their major developmental tasks are decision-making on a career path through education, career success for economic independence, and independence from the parental care through marriage (63). Currently, however, young people in South Korea face a high unemployment rate and an unstable employment environment that, when coupled with various unfavorable socioeconomic conditions, delay the achievement of important life tasks, such as career and marriage (22, 64, 65). This concern be understood in the context of a situation that deprives young people of a sense of belonging, causes conflict with their family, instills a feeling of lacking social respect, prevents them from entering the labor market, which is crucial for forming new social relationships in young adulthood, and results in their social isolation (29, 31, 66, 67). Therefore, young people's social isolation should be interpreted in association with their developmental tasks, achievement level, and identity building.

Second, among the sociopsychological characteristics, relative deprivation, depression, negative future outlook, and low self-esteem were identified as factors that increase social isolation. This outcome supports the findings of previous studies, which showed that relative deprivation, depression, hope, future expectations, and low self-esteem threaten mental health and trigger feelings of helpless (24–27, 68).

Last, among the relationship characteristics, lower social skills, more experience of violence in the past, and less online activity increased social isolation, which support the findings of previous studies (35–37). The abovementioned findings can be seen as a statistical verification of the report of a fact-finding survey on South Korean adolescents (20), which showed that young people with a high degree of social isolation frequently report negative experiences during childhood and adolescence. Furthermore, although the experience of violence was a significant factor at a low significance level (*p* < 0.1) in this study, it can be considered an important risk factor for social isolation because its effect is manifested in an interrelationship with negative emotions. With regard to the influence of online activity, the findings of this study are contradictory to those of some previous studies (1, 69, 70), which showed that online activity increases social isolation. This can be considered from several aspects, including age-dependent relationship characteristics among young people and culture. Thus, the modern-day young adults are familiar with digital relationships to the extent of being called the digital generation. In South Korea, in particular, with the development and ubiquity of the Internet, there are intensive and extensive online activities, such as games, social media, and online meetings, that constitute a routine aspect of daily living. This phenomenon has been further accelerated due to the COVID-19 pandemic, wherein online activities have surged over the past 2 years. Due to social distancing, many face-to-face daily activities have transitioned to virtual video communication platforms, such as Zoom and Skype, and these include communication and sharing of hobbies using social media. Thus, considering this situation and the lifestyle and cultural characteristics of the young generation in South Korea, online activities are closely related to the daily life of young people and can be considered a space and means of communication to form relationships. Therefore, it can be considered that young people may feel disconnected or lonely when they do not undertake many of these activities.

The implications of the important results of this study are as follows. First, to solve the problem of social isolation among young people, customized support systems and community-wide networks should be established, with consideration to the life cycle developmental tasks of the youth, at the national level. For example, there is a need to create a department or delivery system that systematically implement policies with regard to the social isolation problem of the young adults, such as *hikikomori* in Japan, disconnected youth in the United States, and the ministry of loneliness in the United Kingdom. Currently, although South Korean society is aware of the importance of responding to the problem of young people's social isolation, the related services are fragmented and lack systematic implementation. In this regard, it is crucial to set up a delivery system in which public–private partnerships and interactions between the central government, local governments, and local communities form an integrated and effective support infrastructure. In addition, job search programs should be provided to young people who are looking for a job or are unemployed, which adversely affects social isolation. In the context of delay in achieving developmental tasks, various public welfare and social support services need to be provided at the local community level to prevent the affected young people from being alienated from society. Support should be provided in multidimensional life areas, such as jobs, education, housing, living (welfare and health), social participation, and activities, such that young people can be reconnected with society through new opportunities and thus break away from social isolation.

Second, it is necessary to provide the young adults with psychological and emotional support and various mental health services. Relative deprivation, depression, negative future outlook, and self-esteem are different concepts but are risk factors for mental health that can be derived from the realistic situation in which today's young people find themselves. Therefore, in addition to the support related to employment and socioeconomic conditions, diverse mental health services should be provided through preventive and therapeutic interventions, such as resilience promotion, resocialization, and psychological coping skills, to empower young people to cope with adverse situations and to manage the feelings of deprivation, anxiety, and helplessness that they experience from society in a healthy way. Furthermore, young people who have experienced violence in the past should be provided with post-traumatic stress management and intervention programs with a view to prevent social isolation, which can be managed within the framework of long-term monitoring of the progress. In this context, it is necessary to set up a system of early detection of vulnerable young adults and administration of appropriate intervention to prevent social isolation through the abovementioned services.

Lastly, it is necessary to improve accessibility to online and offline mental health services, to take into account the lifestyle and cultural characteristics of young people, and to provide programs related to social and interpersonal skills to promote social relationships in universities, workplaces, and local communities. Additionally, given the trends of increasing contact-free exchanges in the wave of digital transformation in modern society, further review and discussion will be required to determine how far the social networks and emotional relationships can be expanded among young people who are familiar with the digital culture.

The significance of this study is 3-fold. First, this study empirically demonstrated the multidimensional determinants of social isolation, which is a surging global phenomenon in the aftermath of the COVID-19 pandemic. Second, this study investigated the phenomenon of social isolation among young people with regard to both external isolation, which means actual disruption of interpersonal relationships, and internal isolation, which is perceived by young people as feelings of being excluded from the outside world, loneliness, and isolation. Third, the results of the study expand the scope and paradigm of target populations of social isolation by examining young people's social isolation, going beyond the traditional target populations of older adults and single households.

The limitations of this study include the fragmentary cross-sectional study design, as we could not consider the process of social isolation of young people or duration of the state of isolation when explaining the phenomenon of young people's social isolation. Therefore, follow-up studies to examine the pathways to social isolation that factor in the logical associations among variables or to perform a longitudinal analysis will provide richer results for understanding the phenomenon of social isolation among young people.

In conclusion, the difficulties facing the young adults have become multilayered and more serious due to the general economic downturn and social disruption caused by the COVID-19 pandemic. This has amplified the problems of lonely deaths and social isolation among young people in South Korean society. This study examined the physical and subjective dimensions of social isolation among young people (19–39 years) and identified multidimensional risk factors for their social isolation. Higher age and unemployment increase the level of social isolation among young people. Psycho-emotional characteristics, including relative deprivation, depression, low self-esteem, and negative socioeconomic future outlook, were identified as significant risk factors for social isolation. Among the relationship characteristics, social skills, a history of experiencing violence, and online activity were identified as significant factors that affect social isolation. The results of this study highlight the need for various mental health interventions for preventing social isolation as well as a customized public support system that enables the achievement of the life tasks of young people.

## Data availability statement

The original contributions presented in the study are included in the article/supplementary material, further inquiries can be directed to the corresponding author/s.

## Ethics statement

The studies involving human participants were reviewed and approved by Jeonbuk National University Institutional Review Board. The patients/participants provided their written informed consent to participate in this study.

## Author contributions

S-BL, YS, YJ, and SK conceptualized and designed the study. S-BL analyzed the data. S-BL, YS, and SK wrote the first draft and integrated suggestions from all other authors. All authors made significant contributions to the interpretation of the data and drafting of the manuscript, critically revised the manuscript, and approved the final draft.

## Funding

This research was supported by the BK21 FOUR funded by the Ministry of Education (MOE, Korea) and National Research Foundation of Korea (NRF) (4199990314436). Also, BK21 FOUR Program by Jeonbuk National University Research Grant.

## Conflict of interest

The authors declare that the research was conducted in the absence of any commercial or financial relationships that could be construed as a potential conflict of interest.

## Publisher's note

All claims expressed in this article are solely those of the authors and do not necessarily represent those of their affiliated organizations, or those of the publisher, the editors and the reviewers. Any product that may be evaluated in this article, or claim that may be made by its manufacturer, is not guaranteed or endorsed by the publisher.
